# Combining Reversible Electroporation and Bleomycin in Treatment of Arteriovenous Malformations

**DOI:** 10.1007/s00270-025-04178-5

**Published:** 2025-09-12

**Authors:** Florian Obereisenbuchner, Vanessa F. Schmidt, Constantin Goldann, Richard Brill, Daniel Puhr-Westerheide, Elena Borisch, Julia Haehl, Alexandra Hartel, Beate Häberle, Jens Ricke, Max Seidensticker, Melanie A. Kimm, Moritz Wildgruber, Walter A. Wohlgemuth

**Affiliations:** 1 Department of Radiology, LMU University Hospital, LMU Munich, Munich, Germany; 2 Interdisciplinary Center for Vascular Anomalies (IZGA), LMU University Hospital, LMU Munich, Munich, Germany; 3 Clinic and Policlinic of Radiology, Martin-Luther University Halle-Wittenberg, Halle (Saale), Germany; 4 Department of Diagnostic and Interventional Radiology and Neuroradiology, München Klinik Harlaching, Munich, Germany; 5 Department for Pediatric Surgery, LMU University Hospital, LMU Munich, Munich, Germany

**Keywords:** BEST, Bleomycin electrosclerotherapy, BEET, Bleomycin electroembolotherapy, Bleomycin, AVM

## Abstract

**Purpose:**

This study aims to evaluate and compare safety and clinical outcomes of reversible electroporation with either intravenous (BEST) or intraarterial (BEET) Bleomycin application treating extracranial AVMs unsuitable for conventional approaches defined as fine-fistulous AVMs close to vulnerable anatomical structures (such as skin/end-arteries) not amenable for embolization or resection due to inappropriate risk/benefit and/or therapy-refractory or recurrent lesions.

**Materials and Methods:**

This is a sub-analysis of the prospective multicenter APOLLON trial (German clinical trial register, DRKS00021019). Clinical and imaging findings were assessed at baseline and 6-month follow-up to evaluate subjective outcome (symptom-free, partial relief, no improvement, clinical progression) and AVM lesion devascularization on MRI (total, 100%; substantial, 76–99%; partial, 51–75%; slight, 50%; progression). BEST versus BEET was at the discretion of the operator; subgroup outcome comparisons were subsequently performed.

**Results:**

Twenty-one AVM patients received 31 treatments (16/31 BEST, 51.6%; 15/31 BEET, 48.4%); the mean number of procedures per patient was 1.5 (± 0.7). Complications occurred after 7/31 (22.6%) procedures, including 6.4% major complications (delayed wound healing solved by split-skin transplantation, persistant scarring). Subjective outcome revealed partial symptom relief in 13/21 (61.9%) patients, and 4/21 (19.0%) patients presented symptom-free. In 4/21 (19.0%) patients, no improvement or symptom worsening was reported. Imaging revealed complete devascularization in one case (6.3%), substantial (76–99%) and partial (51–75%) devascularization in 6/16 (36.5%) patients, respectively, while progression was noted in 3/16 (18.8%) patients. Comparison of clinical outcomes differed between both approaches, with BEET being superior to BEST (*p* = 0.04).

**Conclusion:**

The combination of reversible electroporation and bleomycin is effective for treatment of AVMs; BEET tends to present superior to BEST regarding patients ’ outcome.

**Supplementary Information:**

The online version contains supplementary material available at 10.1007/s00270-025-04178-5.

## Introduction

Recently, Bleomycin Electrosclerotherapy (BEST) has been developed as a novel therapeutic method for treating slow-flow vascular malformations [1]. After intravenous or intralesional injection of Bleomycin, reversible electroporation induces a temporary increase in cellular membrane permeability, whereby the intracellular concentration of Bleomycin increases. This may increase the efficacy of Bleomycin [2], and initial promising results have been described in first studies of slow-flow malformations [3]. BEST in high-flow lesions may be a valuable alternative in lesions where embolization or surgery cannot be performed with a reasonable risk–benefit ratio [4–6]. The aim of this study was to prospectively evaluate safety and short-term outcome of AVMs treated by a combination of reversible electroporation and bleomycin and to compare systemic intravenous bleomycin application (BEST) to intraarterial bleomycin application (BEET) in lesions either refractory or not amenable to conventional treatment.

## Materials and Methods

### Study Design, Follow-Up, and Outcome Assessment

This study is a subanalysis of the prospective multicenter APOLLON trial (German clinical trial register, DRKS00021019, protocol no. 20–445) which investigates various treatment options including conservative management, medical therapy, minimally invasive image-guided procedures, surgery, and their combinations [7]. For this subanalysis, all patients with symptomatic AVMs treated with BEET or BEST between March 2021 and May 2024 were included. Study and procedural details are provided in the Supplementary.

### Statistical Analysis

Descriptive statistics were used to analyze the distribution of variables among the different categories. Kolmogorov–Smirnov test was used for assessment of normality. Data are presented as mean (± standard deviation) or median (range, minimum–maximum). Subgroup comparisons were performed using Pearson’s Chi-squared test for categorical data. Statistical testing was conducted using SPSS (version 26.0, IBM Corp., USA); *p *< 0.05 was considered significant.

## Results

### Patients Characteristics

**Fig. 1:**
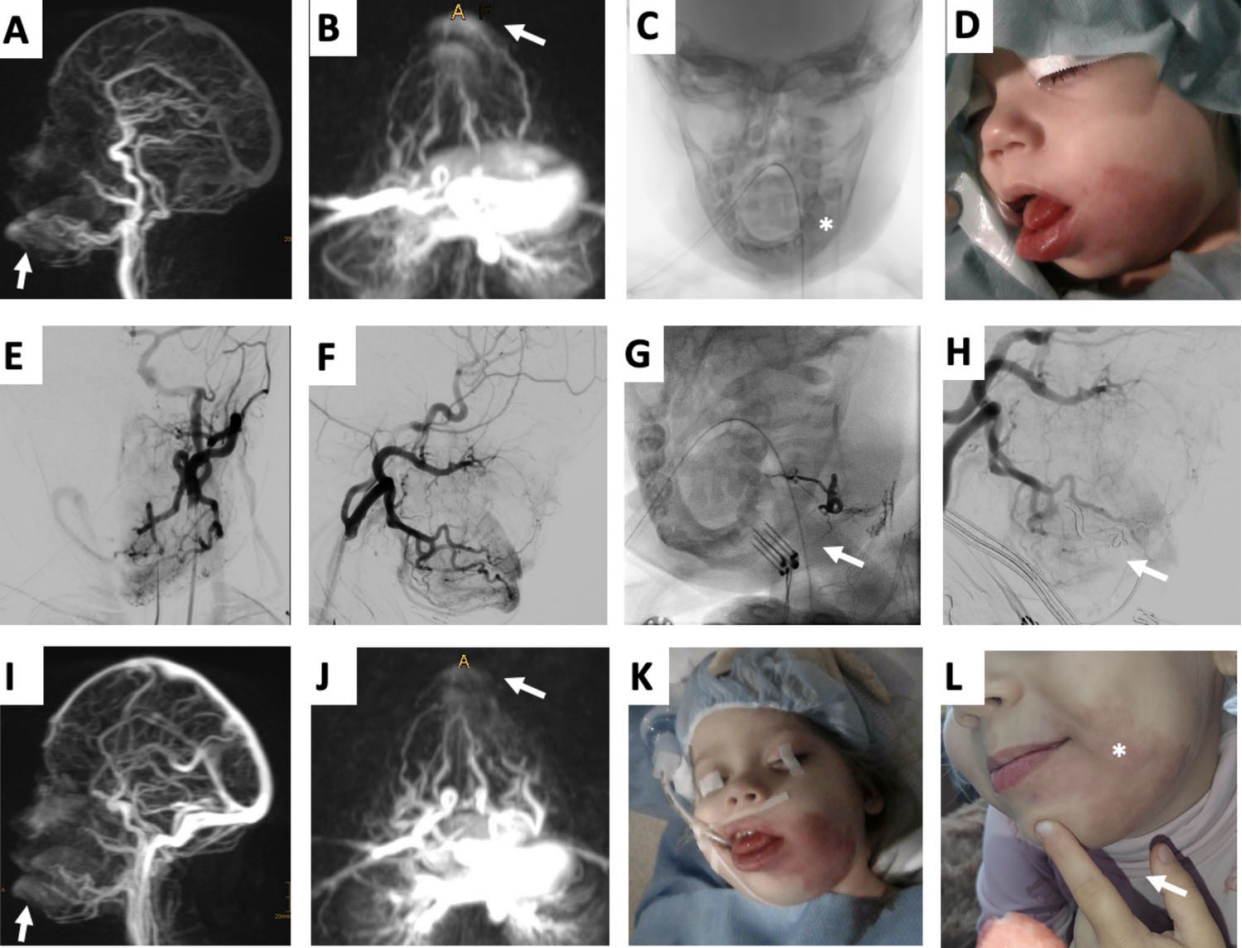
One-year-old female patient with capillary malformation-arteriovenous malformation (CM-AVM) syndrome presenting CMs on various localizations, an AVM on the left cheek as well as an osseous hyperplasia of the left mandibula undergoing two sessions of Bleomycin Electrosclerotherapy (BEST). **A, B,** MR-angiography presenting relevant fast-flow shunting of the vascular malformation on the left cheek involving tongue, gingiva, and lips (arrows). **C,** Digital subtraction angiography (DSA) image demonstrates an accompanying osseous hyperplasia of the left mandible (asterisk). **D,** Clinical presentation of the patient prior to treatment including noticeable swelling of the affected region and pink discoloration of the skin due to the arterial component in this lesion. **E, F,** DSA images before BEST revealing detailed vasculature architecture including angiographic classification (Cho type IIIa). **G, H,** DSA image during BEST procedure verifying electrode placement (arrow, I) as well as after BEST presenting immediately reduced AVM perfusion (H). **I, J,** MR-angiography showing reduced fast-flow shunting of the AVM (arrows) compared to A and B. **K,** Clinical presentation of the patient immediately after the second session of BEST at the age of two years. **L,** Clinical presentation of the patient at the age of three years, note the significantly reduced swelling and weakened pink discoloration

**Fig. 2:**
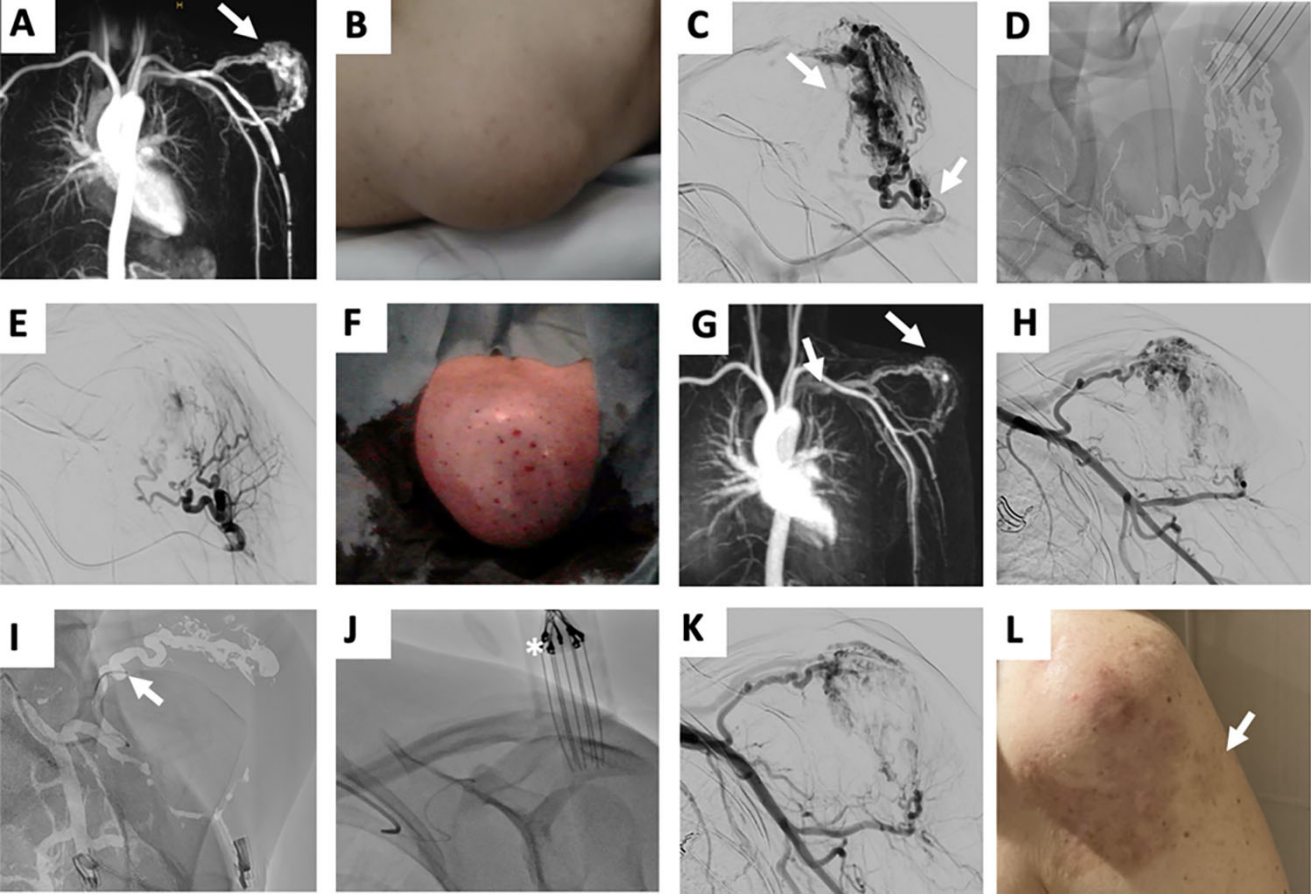
Thirty-nine-year-old female patient with arterio-venous malformation (AVM) of shoulder undergoing two sessions of Bleomycin Electroembolotherapy (BEET). **A,** MR-angiography presenting location and extension of the vascular malformation on the left shoulder involving relevant fast-flow shunting (arrow). **B,** Clinical presentation of the patient prior to treatment including subtle pink discoloration of the skin. **C,** Digital subtraction angiography (DSA) demonstrates the vascular architecture of the lesion and microcatheter placement immediately before intra-arterial application of Bleomycin (arrow). **D,** DSA image during electroporation performed synchronously to embolotherapy. **E,** DSA image straight after BEET presenting significantly reduced lesion perfusion due to vascular lock phenomena. **F,** Clinical presentation immediately after BEET. **G,** MR-angiography three months after first BEET showing reduced fast-flow shunting of the AVM (arrow) compared to A. **H,** DSA image before admitting second BEET, notice the devascularization compared to C. **I, J,** DSA images during second BEET procedure verifying catheter (arrow) and electrode (asterisk) placement. **K,** Postprocedural DSA image demonstrates further reduced lesion perfusion. **L,** Clinical presentation of the patient one year after the second BEET, with slight postprocedural skin discoloration on the electroporation site (arrow) persisting at 6 months

Twenty-one patients with extracerebral AVMs (9 males, 12 females) underwent a total of 31 BEST/BEET procedures (Supplemental Table 1). Seventeen (81.0%) patients were part of the prospective multicenter study APOLLON; four (19.0%) patients were ineligible due to age limitation (≤ 4 years) but received similarly structured follow-up. The median age was 33 years (range, 0.5–53 years) at treatment. Twelve (57.1%) AVMs involved the face, with 5/21 (23.8%) lesions including lips (Fig. 1 and Supplemental Fig. 1), 4/21 (19.0%) enoral areas, and 1/21 (4.8%) periorbital region. Furthermore, 7/21 (33.4%) AVMs were located along the extremities and 3/21 (14.3%) AVMs on the trunk (Fig. 2). Cho’s classification [8] showed mostly type IIIa (11/19, 57.9%) and type IIIb (8/19, 42.1%). Regarding Schobinger classification [9], 2/21 (9.5%) were categorized as stage 2, 18/21 (85.7%) patients as stage 3, and 1/21 (4.8%) as stage 4. Eleven (52.4%) patients had undergone previous treatment, including incomplete embolization (11/21, 52.4%) and/or partial surgical resection (7/21, 33.3%).

**Table 1:** Comparison of BEST vs. BEET in AVMs

Characteristic	Total cohort (n = 21/31/21/16)	BEST (n = 10/16/10/9)	BEET (n = 11/15/11/7)	*p*-value
*Baseline schobinge*				*p* = 0.55^a^
Stage 1	0/21 (0.0%)	0/10 (0.0%)	0/11 (0.0%)	
Stage 2	2/21 (9.7%)	1/10 (10.0%)	1/11 (9.1%)	
Stage 3	19/21 (85.7%)	8/10 (80.0%)	10/11 (90.9%)	
Stage 4	1/21 (4.8%)	1/10 (10.0%)	0/11 (0.0%)	
*Procedural details*				
Bleomycin dose, mean (± SD)	8.9 (± 5.9)	10.6 (± 5.2)	7.1 (± 6.2)	*p* = 0.10^b^
Electroporation cycles, mean (± SD)	20.6 (± 13.1)	23.1 (± 15.0)	17.9 (± 10.8)	*p* = 0.28^b^
*Clinical outcome*				*p *= 0.04^a^
Symptom-free	4/21 (19.0%)	0/10 (0.0%)	4/11 (36.4%)	
Partial relief	13/21 (61.9%)	6/10 (60.0%)	7/11 (63.6%)	
No improvement	2/21 (9.5%)	2/10 (20.0%)	0/11 (0.0%)	
Progression	2/21 (9.5%)	2/10 (20.4%)	0/11 (0.0%)	
*Imaging outcome*				
Complete devascularization	1/16 (6.3%)	0/9 (0.0%)	1/7 (14.3%)	*p* = 0.28^a^
Substantial devascularization	6/16 (37.5%)	3/9 (33.3%)	3/7 (42.9%)	
Partial devascularization	6/16 (37.5%)	3/9 (33.3%)	3/7 (42.9%)	
No devascularization	0/16 (0.0%)	0/9 (0.0%)	0/7 (0.0%)	
Progression	3/16 (18.8%)	3/9 (33.3%)	0/7 (0.0%)	

*AVM* = Arteriovenous malformation, *Beet* = Bleomycin electroembolotherapy, *Best* = Bleomycin electrosclerotherapy, *SD* = standard deviation; *Vs* = versus, ^a^Pearson’s Chi-squared, ^b^Unpaired *t* test

### Procedural Characteristics

Thirteen (61.9%) patients received one BEST/ BEET procedure, 6/21 (28.6%) patients received two, and 2/21 (9.5%) patients received three procedures (mean 1.5 ± 0.7 procedures). The mean number of electroporation cycles per treatment was 20.6 ± 13.1, in the BEST subgroup 23.1 ± 15.0, and in the BEET subgroup 18.0 ± 10.8. The mean dose of bleomycin was 8.9 ± 5.9 mg per session, in the BEST subgroup 10.6 ± 5.2 mg, and in the BEET subgroup 7.1 ± 6.2 mg (Supplemental Table 2).

### Safety and Complications

Postprocedural complications were reported after 7/31 (22.6%) procedures (grade 2–3), including excessive swelling at the electroporation site (4/31, 12.9%, grade 2), postprocedural hematoma followed by delayed wound healing, solved by split-skin transplantation (1/31, 3.2%, grade 3), and persistent scarring in the treated area on the cheek not requiring surgery (1/31, 3.2%, grade 4). One patient with a facial AVM demonstrated jaw pain when chewing after BEET, completely regressing within 6 months without any additional measures (1/31, 3.2%, grade 2). Postprocedural skin discoloration limited to the electroporation site occurred after 14/31 (45.2%) procedures, in all cases fading in the postprocedural course though still visible at 6-months follow-up.

### Outcome

Thirteen (61.9%) patients showed partial relief of symptoms, 4/21 (19.0%) patients presented symptom-free, 2/21 (9.5%) without improvement of symptoms, and 2/21 (9.5%) with clinical progression (in both cases confirmed by MRI). MRI was available in 16/21 patients. In children ≤ 4 years (*n *= 4) of age, whose lesions were easy to monitor by ultrasound, no MRI was performed at follow-up. Substantial devascularization (76–99%) and partial devascularization (51–75%) were assessed in 6/16 patients (37.5%), respectively. Progression of the lesion was found in 3/16 patients (18.8%) and complete devascularization in 1/16 patients (6.3%).

### Subgroup Comparison of BEST vs. BEET

Ten (47.6%) patients were treated by 16/31 (51.6%) BEST procedures, 11/21 (52.4%) patients by 15/31 (48.4%) BEET procedures (Table 1). The comparison of clinical outcome differed between both subgroups (*p *= 0.04); exemplarily, 4/11 (36.4%) patients treated by BEET rated symptom-free, while 0/10 (0.0%) patients treated by BEST did so. There were no significant differences in lesion devascularization on MRI (*p *= 0.28), though progression was only observed after BEST (3/9, 33.3%).

## Discussion

AVMs located in vulnerable areas, such as the face or those involving skin infiltration, were previously considered not amenable by either interventional or surgical methods due to the high risk of tissue necrosis. Although first targeted medical approaches showed success in either stopping progression or at best lesion reduction [10], those therapies come with severe side effects, and it remains unclear if effects persist after discontinuation. Thus, alternative treatments are needed for both difficult anatomic locations and AVMs refractory to conventional therapies.

Electrochemotherapy (ECT) is effective in bleeding tumor lesions [11]. The effect of combining reversible electroporation and bleomycin is attributed to a combination of potentiated local cytotoxic and anti-vascular effects of bleomycin and electroporation [12–14]. The effects include an acute “vascular lock” effect, essentially being a high-voltage induced vasospasm [15], preventing bleeding and reducing bleomycin wash-out. Additionally, longer lasting effects of intracellular bleomycin (vascular disrupting effect) on dysplastic vascular endothelial cells induce regression [14]. Simultaneously, cytotoxic effects on the respective endothelium reduce pro-angiogenic signaling in the treated AVM [1], potentially limiting proliferation or recurrence.

Considering the combination of reversible electroporation and Bleomycin for the treatment of AVMs, this study reports a new indication adding on to first preliminary results [4–6] demonstrating an acceptable safety profile and effectivity in the treatment of AVMs. Subjective outcomes differed between BEST and BEET, with BEET yielding more symptom-free patients and no non-responders or cases of progression. Both subgroups demonstrated a high percentage of either partial or substantial devascularization. The differing responses may be explained by higher local intravascular concentration of Bleomycin at the endothelium during first pass following intraarterial administration. BEET, however, requires selective catheterization of the AVM. An advantage of combining the procedure with DSA is improved understanding of AVM vascular anatomy, allowing for more precise targeting during electroporation. Bouwman reported > 69% devascularization in 69% of ethanol-embolized AVMs, whereas 81% of our cohort achieved > 51% devascularization [16]. However, direct comparison is limited due to relevant differences in lesion types, with our study predominantly containing fine-fistulous type IIIa AVMs versus mainly type II lesions in Bouwman’s cohort. Prospective studies directly comparing techniques in AVMs of equivalent angioarchitecture are warranted.

Important limitations of our study are cohort size and lack of long-term outcome data beyond 6 months. As there were no objective selection criteria of BEST versus BEET, the initial trend of superior outcomes with BEET should be confirmed through randomized study protocols. BEST/BEET procedures are not yet standardized, and guidelines will have to be developed. Additionally, outcome differences between BEST and BEET may reflect selection bias and a trend toward performing BEET later in the study, thus at a later stage along the learning curve. Another line of clinical research will be dose optimization schemes and comparison to interstitial [6, 17] administration of Bleomycin prior to reversible electroporation. Similarly, objective imaging-based response criteria for AVM devascularization must be established.

Overall, this study demonstrated that bleomycin combined with reversible electroporation is an effective treatment for peripheral fast-flow vascular malformations, with most peri- and postprocedural complications healing without sequelae.

## Supplementary Information

Below is the link to the electronic supplementary material.

Supplementary file1 (DOCX 530 kb)

## Abbreviations

AE: Adverse event
AVM: Arteriovenous Malformation
BEET: Bleomycin Electroembolotherapy
BEST: Bleomycin Electrosclerotherapy
CIRSE: Cardiovascular and Interventional Radiological Society of Europe
DSA: Digital Subtraction Angiography
ECT: Electrochemotherapy
ia.: Intraarterial
iv.: Intravenous
LEA: Liquid embolization agents
MRI: Magnetic resonance imaging
PACS: Picture Archiving and Communication System
RAS: Rat sarcoma
T1w: T1-weighted
T2w: T2-weighted
VAS: Visual Analog Scale
vs: Versus
WMA: World Medical Association
