# Supplementary material for: Combining Reversible Electroporation and Bleomycin in Treatment of Arteriovenous Malformations
Source: Cardiovasc Intervent Radiol. 2025 Sep 12;48(11):1632–7. doi: 10.1007/s00270-025-04178-5 (PMC12571998; doi:10.1007/s00270-025-04178-5)
Supplement: Supplementary file 1 — Supplementary file1 (DOCX 530 kb) [file 270_2025_4178_MOESM1_ESM.docx]

*Study cohort*

Patients have been recruited to the APOLLON protocol consecutively via the corresponding interdisciplinary vascular anomalies centers at each institution. The following inclusion and exclusion criteria applied according to the study protocol.

Inclusion criteria:

- Age >4 years, <70 years
- Patients with simple peripheral (=extracranial) arteriovenous malformations (AVMs) according to the ISSVA (International Society for the Study of Vascular Anomalies) classification. Each patient and the corresponding diagnosis, is discussed in an interdisciplinary board for vascular anomalies before initiation of treatment.
- Patients with combined vascular malformations and AVMs associated with other anomalies (e. g. Parkes Weber, PTEN hamartoma, HHT) according to the ISSVA (International Society for the Study of Vascular Anomalies) classification
- Patients with first line therapy or patients with previous alternative therapies in whom the previous treatments did not lead to durable symptom improvement
  - Previous surgery or embolization are not an exclusion criterion, but these patients will only be included in case of a therapy-free interval of 12 months

Exclusion criteria:

- Patients with AVMs located in the central nervous system
- Patients with AVMs located in abdominal parenchymal organs and the gastrointestinal tract
- Patients with other high-flow vascular anomalies (e. g. vascular tumors)
- Patients who have been previously treated by surgery or sclerotherapy/embolization within the last 12 months
- Patients with concomitant life-limiting diseases (such as cancer)
- Patients with acute inflammatory diseases or acute bacterial superinfection of an AVM related ulceration
- Patients with contraindications for invasive treatments
  - Patients with contrast agent intolerance or renal insufficiency (GFR>30ml/min)
  - Patients with impaired coagulation status:
    - Platelet count <50.000/μl
    - aPTT >50s
    - INR >1,5
  - Poor general condition with ECOC performance > 1
- Pregnant or breast-feeding women
- Inability to access the AVM lesion due to anatomical or pathoanatomical reason
- Inability of the patient/parents to understand or follow the study protocol e.g. due to impaired mental health status
- Patients/parents who refuse to give informed consent

Indications for BEST/BEET were established following interdisciplinary consensus at two Interdisciplinary Vascular Anomalies Centers and were based on clinical symptoms (pain, pulsation, swelling, bleeding, ulceration), aesthetic disfigurement and functional impairment as well as after lesion assessment on contrast enhanced MR Imaging. Selection criteria for treatment with reversible electroporation were: Firstly, lesions not considered amenable for conventional treatments such as embolization or surgery, fine-fistulous AVMs (Cho type IIIa) close to the skin [1], AVMs with skin infiltration, AVMs along end-arteries such as the fingers and AVMs close (<1cm) to vulnerable anatomical structures (major vessels/nerve bundles), and secondly, therapy-refractory or recurrent lesions, defined as lesions that persist or progress either clinically or radiologically or both after previous embolization or surgery. All lesions presented at superficial localizations less than 5 cm deep, readily accessible for electroporation. Initial cases preferentially had AVM skin infiltration and were treated with intravenous applied Bleomycin, comparable to the established method of bleomycin electrochemotherapy [2] for skin tumors and little modified to the two published case reports [3, 4]. With experience gained, additional deeper situated lesions were included, and the method was varied to include intraarterial bleomycin application. However, there was no clear-cut differential indication for the BEST versus BEET approach. In general, BEST/BEET was not performed in breastfeeding or pregnant women, patients with childbearing potential not using contraception, patients with intolerance to Bleomycin or previous Bleomycin-related toxicity, patients who already received a cumulative dose of Bleomycin of >100 mg, patients with known pulmonary dysfunction, patients with previous chest radiation therapy, and patients with a history of epilepsy/seizures [2, 5, 6].

*Follow-Up*
Patients were scheduled for a standardized follow-up at 6-months as described in the protocol [7]. In case of insufficient improvement of symptoms and residually vascularized lesion being present, additional treatment was performed. Subsequent follow-up visits including MR imaging were scheduled on an annual basis thereafter.

*Peri- and postprocedural complications*
All peri- and post-procedural complications were classified into minor and major according to the Cardiovascular and Interventional Radiological Society of Europe (CIRSE) classification system [8]. Special attention was paid to document skin alterations (necrosis, blebs), and peripheral nerve injuries. Commonly occurring postprocedural skin discoloration was evaluated additionally.

*Outcome*
Clinical response at follow-up was measured by the following grading scale: symptom-free, partial relief of symptoms, no improvement of symptoms, and clinical progression despite treatment. Objective response was assessed by changes in AVM devascularization using pre-procedural MR images compared to follow-up after the last BEST/BEET treatment. Imaging findings were divided into 5 categories: total devascularization (100%), substantial remission (76–99%), partial remission (51–75%), slight remission (<50%), and progression. These examinations were evaluated in consensus by two radiologists (FO, VFS) with 4 and 7 years of experience in vascular MRI.

*Bleomycin Electroembolotherapy (BEET) and Bleomycin Electrosclerotherapy (BEST)*

Initially, patients were first treated with intravenous Bleomycin application, which is the established application mode for slow-flow malformations, and later modified to intraarterial Bleomycin application directly into the nidus via a microcatheter. None of the patients with multiple procedures received both technical approaches.
Interventional treatment was performed under general anesthesia. Intravenous or intraarterial injection of Bleomycin was followed by electrode positioning and subsequent application of reversible electroporation pulses [6, 9]. Intravenous Bleomycin was given via a cubital venous access, with a concentration of 1 mg Bleomycin dissolved in 1 ml NaCl. For intraarterial Bleomycin application, a coaxial microcatheter was placed close to the AVM nidus, in case of multiple feeders the dominant feeding artery was chosen.

Needles were placed within the margins of the malformations at distances ranging from 0.5 to 3 cm. Thus, the target volume was completely covered side-by-side with repetitive punctures with electric field overlapping being avoided. The dose of Bleomycin administered was chosen according to both the size of the lesion and the age and weight of the patient, however it is important to mention that no standardized application schemes exist for Bleomycin dosing in this setting. The maximum overall Bleomycin dose allowed was 0.2 mg per kg bodyweight per treatment session and less than 1 mg/kg bodyweight cumulative according to standard Bleomycin sclerotherapy, which is less compared to previous reports [2, 6]. To apply the reversible electric pulses, the electroporation system (Cliniporator™ VITAE, IGEA S.p.A., Carpi, Italy) was used, which provides independently controlled and isolated outputs each reaching up to 3000 V (maximum current: 50 A) per cm. Consequently, electrical pulses with a duration of 100 μs between each pair of electrodes were generated. Number and intensity of electric pulses for electroporation are calculated by the pulse generator without the possibility for manual adjustments in order to achieve optimal heterogeneity of the field for reversible electroporation while at the same time limiting the amount of electric energy deposits in order to avoid irreversible electroporation and tissue damage. The electroporation was performed either 1 minute after starting the bleomycin injection via a perfusion pump at 1mg/min in case of intraarterial administration or 8 minutes after starting intravenous Bleomycin administration. Eight minutes were chosen according to the pharmacokinetics of bleomycin in order to allow enough time for the bleomycin to distribute and accumulate at the target site. Although not significant, the applied bleomycin dose was slightly lower in the BEET group for intraarterial application, as we assumed high enough tissue concentrations via this local delivery approach. Of note, electroporation was similar between BEET and BEST without differences in pulse frequency or intensity. Intraarterial selective digital subtraction angiograms were obtained before and after completing electroporation.


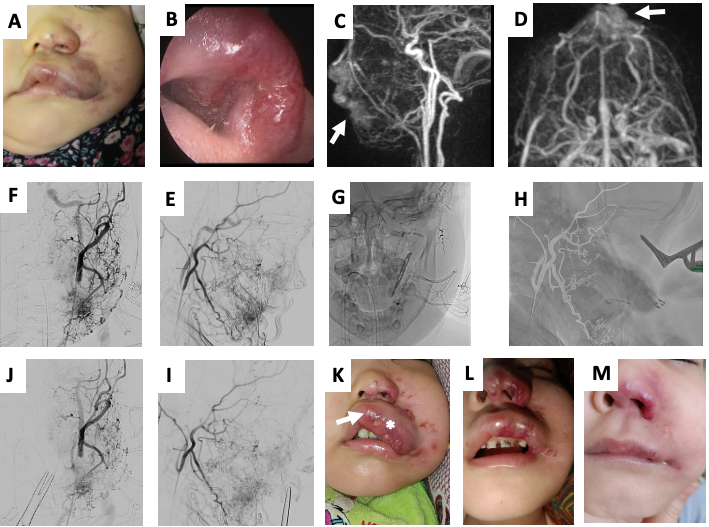


**Supplemental Figure 1.**

**Four-year-old female patient with arteriovenous malformation (AVM) of the face undergoing one Bleomycin Electroembolotherapy (BEET) after history of 8 transarterial embolizations without sufficient symptom improvement and complicative blinding of the right eye after embolization with liquid embolization agent. A,** Clinical presentation of the patient prior to treatment including noticeable swelling of the affected region left-sided (lips, nose, cheek) with consecutive mouth asymmetry, skin alterations and recurrent (weekly) splashing epistaxis. **B,** Rhinoscopy revealed bleeding foci in the nasal vestibule on the inner wing of the left nose. **C, D,** MR-angiography presenting relevant fast-flow shunting of the AVM (arrows). **E, F,** Digital subtraction angiography (DSA) images before BEET revealing detailed vasculature architecture. **G, H,** Periprocedural DSA images presenting AVM marking (G) and transarterial Bleomycin application during reversible electroporation (H). **I, J,** DSA images after BEET revealing detailed reduced lesion vascularization. **K,** Clinical presentation immediately after BEET with postinterventional swelling (not above average, asterisk) and skin alterations such as small necroses and blebs (arrow). **L,** Continuously decreasing swelling and good healing of necrosis and blebs over time. **M,** Clinical presentation a few weeks after BEET while epistaxis was completely resolved and nearly total regredient swelling of the affected areas.

**Supplemental Table 1. Patient characteristics and clinical presentation of study cohort.**

| **Patient No.** | **Age at inclusion** | **Sex** | **Type of procedure** | **No. of**  **cycles** | **Symptoms** | **Lesion localization** | **Angiographic**  **classification**  **(Cho^a^)** | **Schobinger**  **classification^b^** | **Previous**  **treatment** | **Clinical**  **outcome** | **VAS pain**  **score**  **before**  **therapy** | **VAS pain**  **score**  **after**  **therapy** |
| --- | --- | --- | --- | --- | --- | --- | --- | --- | --- | --- | --- | --- |
| **1** | **28** | **f** | **BEST** | **3** | **pulsation, swelling, pain, ulceration, functional impairement, high-output cardiac failure** | **face, neck, UE (upper arm)** | **IIIB** | **4** | **embo.** | **partial relief** | **7** | **3** |
| **2** | **48** | **f** | **BEST** | **2** | **swelling, pain, skin alterations** | **trunk** | **IIIB** | **3** | **none** | **partial relief** | **6** | **3** |
| **3** | **48** | **m** | **BEST** | **2** | **swelling, pain, functional impairement, skin alterations** | **trunk, UE (upper arm, forearm)** | **IIIB** | **3** | **embo.** | **partial relief** | **7** | **4** |
| **4** | **30** | **m** | **BEST** | **1** | **swelling** | **LE (lower leg)** | **n.a.** | **2** | **embo., surgery** | **no improvement** | **0** | **0** |
| **5^c^** | **1** | **f** | **BEST** | **2** | **swelling, pain, functional impairement, skin alterations** | **face (+lips, +enoral)** | **IIIA** | **3** | **none** | **partial relief** | **7** | **3** |
| **6^c^** | **0** | **m** | **BEST** | **1** | **swelling, pain, functional impairement, bleeding, skin alterations** | **face (+lips, +enoral)** | **IIIA** | **3** | **none** | **partial relief** | **5** | **3** |
| **7** | **53** | **f** | **BEST** | **1** | **pulsation, swelling, pain, ulceration, functional impairement, skin alterations** | **UE (forearm)** | **IIIB** | **3** | **embo., surgery** | **no improvement** | **7** | **7** |
| **8** | **50** | **m** | **BEST** | **2** | **swelling, pain, ulceration, skin alterations** | **LE (lower leg, foot)** | **IIIB** | **3** | **embo., surgery** | **partial relief** | **7** | **3** |
| **9** | **34** | **f** | **BEST** | **1** | **swelling, pain, ulceration, skin alterations** | **UE (forearm)** | **IIIB** | **3** | **none** | **progression** | **7** | **8** |
| **10^c^** | **1** | **f** | **BEST** | **1** | **swelling, pain, functional impairement, bleeding** | **face (+lips)** | **IIIA** | **3** | **none** | **progression** | **4** | **5** |
| **11** | **39** | **f** | **BEET** | **2** | **swelling, pain** | **trunk, UE (upper arm)** | **IIIA** | **3** | **none** | **partial relief** | **8** | **1** |
| **12** | **37** | **f** | **BEET** | **1** | **swelling, pain** | **face** | **IIIA** | **3** | **none** | **partial relief** | **3** | **1** |
| **13** | **33** | **m** | **BEET** | **3** | **swelling, pain, ulceration, functional impairement, skin alterations** | **face** | **IIIB** | **3** | **none** | **partial relief** | **5** | **2** |
| **14^c^** | **4** | **m** | **BEET** | **1** | **swelling, pain, bleeding, skin alterations** | **face (+lips, +enoral)** | **IIIA** | **3** | **embo** | **symptom-free** | **3** | **0** |
| **15** | **18** | **m** | **BEET** | **1** | **swelling, pain, ulceration, bleeding, skin alterations** | **LE (thigh, knee, lower leg)** | **n.a.** | **3** | **none** | **partial relief** | **6** | **2** |
| **16** | **13** | **f** | **BEET** | **1** | **swelling, skin alterations** | **face** | **IIIA** | **3** | **embo.** | **partial relief** | **0** | **0** |
| **17** | **29** | **m** | **BEET** | **2** | **pulsation, swelling, pain, ulceration, functional impairement, bleeding, skin alterations** | **face (+periorbital)** | **IIIA** | **3** | **embo., surgery** | **partial relief** | **6** | **2** |
| **18** | **34** | **f** | **BEET** | **1** | **pulsation, swelling, pain, tinnitus** | **face (+enoral)** | **IIIA** | **3** | **embo., surgery** | **symptom-free** | **0** | **0** |
| **19** | **35** | **f** | **BEET** | **1** | **swelling, pain** | **face (+lips)** | **IIIA** | **3** | **none** | **symptom-free** | **4** | **0** |
| **20** | **42** | **f** | **BEET** | **1** | **pulsation, swelling, pain, bleeding** | **face** | **IIIB** | **3** | **embo., surgery** | **partial relief** | **5** | **2** |
| **21** | **46** | **m** | **BEET** | **1** | **pulsation** | **face** | **IIIA** | **2** | **embo., surgery** | **symptom-free** | **0** | **0** |

**BEET=Bleomycin Electroembolotherapy; BEST=Bleomycin Electrosclerotherapy; embo.=embolization; LE=lower extremity; m=man; n.a.=not available; No.=number; UE=upper extremity; w=woman; VAS=Visual Analog Scale; ^1^Cho classification according to Cho et al. (10); ^b^Schobinger classification according to Kohout et al. (11) ; ^c^Patients have not been included into the APOLLON study due to exclusion criteria (age ≤4).**

**Supplemental Table 2. Procedural characteristics and complications of study cohort.**

| **Procedure No.** | **Patient No.** | **Age at treatment** | **Type of procedure** | **Bleomycin dose (mg)** | **Type of**  **electrodes** | **Length of**  **electrodes**  **(mm)** | **Electroporation**  **cycles** | **Post-**  **procedural complication** | **Kind of complication** | **Postprocedural  hyper- pigmentation** |
| --- | --- | --- | --- | --- | --- | --- | --- | --- | --- | --- |
| **1** | **1** | **28** | **BEST** | **10** | **hexagonal** | **15** | **17** | **no** |  | **no** |
| **2** | **1** | **28** | **BEST** | **10** | **hexagonal** | **25** | **18** | **no** |  | **no** |
| **3** | **1** | **29** | **BEST** | **15** | **hexagonal** | **20** | **19** | **no** |  | **no** |
| **4** | **2** | **48** | **BEST^a^** | **12.5** | **hexagonal** | **15** | **30** | **no** |  | **no** |
| **5** | **2** | **49** | **BEST** | **15** | **hexagonal** | **10** | **18** | **no** |  | **no** |
| **6** | **3** | **48** | **BEST** | **15** | **hexagonal** | **20** | **70** | **no** |  | **no** |
| **7** | **3** | **48** | **BEST** | **15** | **hexagonal** | **20** | **45** | **no** |  | **no** |
| **8** | **4** | **30** | **BEST** | **15** | **hexagonal** | **10** | **23** | **no** |  | **yes** |
| **9** | **5^c^** | **1** | **BEST^b^** | **1.5** | **finger** | **15** | **15** | **no** |  | **no** |
| **10** | **5^c^** | **2** | **BEST** | **2** | **finger** | **15** | **14** | **no** |  | **yes** |
| **11** | **6^c^** | **0** | **BEST^b^** | **7.5** | **finger** | **10** | **16** | **no** |  | **no** |
| **12** | **7** | **53** | **BEST** | **15** | **finger** | **15** | **17** | **no** |  | **no** |
| **13** | **8** | **50** | **BEST** | **10** | **finger** | **10** | **26** | **no** |  | **yes** |
| **14** | **8** | **52** | **BEST** | **15** | **hexagonal** | **15** | **14** | **no** |  | **yes** |
| **15** | **9** | **34** | **BEST** | **10** | **hexagonal** | **10** | **16** | **yes** | **Hematoma, delayed wound healing, resolved by split-skin transplantation** | **no** |
| **16** | **10^c^** | **1** | **BEST^b^** | **1** | **finger** | **15** | **11** | **no** |  | **yes** |
| **17** | **11** | **39** | **BEET** | **0.8** | **hexagonal** | **15** | **13** | **no** |  | **yes** |
| **18** | **11** | **39** | **BEET** | **2** | **hexagonal** | **15** | **15** | **no** |  | **no** |
| **19** | **12** | **37** | **BEET** | **0.4** | **freely positionable needle** | **15** | **4** | **no** |  | **yes** |
| **20** | **13** | **33** | **BEET** | **3** | **hexagonal** | **20** | **15** | **no** |  | **no** |
| **21** | **13** | **33** | **BEET** | **2** | **hexagonal** | **20** | **12** | **no** |  | **no** |
| **22** | **13** | **33** | **BEET** | **5** | **hexagonal** | **20** | **16** | **no** |  | **yes** |
| **23** | **14^c^** | **4** | **BEET** | **2** | **finger** | **15** | **16** | **no** |  | **yes** |
| **24** | **15** | **18** | **BEET** | **4** | **hexagonal** | **10** | **27** | **no** |  | **no** |
| **25** | **16** | **13** | **BEET** | **2** | **finger** | **15** | **7** | **no** |  | **yes** |
| **26** | **17** | **29** | **BEET** | **15** | **finger** | **15** | **35** | **yes** | **Prolonged/excessive swelling** | **yes** |
| **27** | **17** | **29** | **BEET** | **15** | **hexagonal** | **20** | **40** | **yes** | **Prolonged/excessive swelling** | **yes** |
| **28** | **18** | **34** | **BEET** | **10** | **finger** | **20** | **30** | **yes** | **Prolonged/excessive swelling** | **yes** |
| **29** | **19** | **35** | **BEET** | **15** | **finger** | **20** | **22** | **yes** | **Prolonged/excessive swelling** | **no** |
| **30** | **20** | **42** | **BEET** | **15** | **hexagonal** | **20** | **5** | **yes** | **Scarring and skin retraction** | **yes** |
| **31** | **21** | **46** | **BEET^a,b^** | **15** | **finger** | **15** | **12** | **yes** | **Jaw pain when chewing, completely resolved with no additional measures** | **no** |

BEET=Bleomycin Electroembolotherapy; BEST=Bleomycin Electrosclerotherapy; n.a.=not available, No.=number; ^a^Additional coil embolization during the same procedure. ^b^Additional ethylene-vinyl-ethanol embolization during the same procedure. ^c^Patients have not been included into the APOLLON study due to exclusion criteria (age ≤4).

**References**

1. Cho SK, Do YS, Shin SW, Kim DI, Kim YW, Park KB, et al. Arteriovenous malformations of the body and extremities: analysis of therapeutic outcomes and approaches according to a modified angiographic classification. J Endovasc Ther. 2006;13(4):527-38. Epub 2006/08/25. doi: 10.1583/05-1769.1. PubMed PMID: 16928170.

2. Gehl J, Sersa G, Matthiessen LW, Muir T, Soden D, Occhini A, et al. Updated standard operating procedures for electrochemotherapy of cutaneous tumours and skin metastases. Acta Oncol. 2018;57(7):874-82. Epub 2018/03/27. doi: 10.1080/0284186x.2018.1454602. PubMed PMID: 29577784.

3. Mir LM, Gehl J, Sersa G, Collins CG, Garbay J-R, Billard V, et al. Standard operating procedures of the electrochemotherapy: Instructions for the use of bleomycin or cisplatin administered either systemically or locally and electric pulses delivered by the CliniporatorTM by means of invasive or non-invasive electrodes. European Journal of Cancer Supplements. 2006;4(11):14-25. doi: 10.1016/j.ejcsup.2006.08.003.

4. Krt A, Cemazar M, Lovric D, Sersa G, Jamsek C, Groselj A. Combining superselective catheterization and electrochemotherapy: A new technological approach to the treatment of high-flow head and neck vascular malformations. Front Oncol. 2022;12:1025270. Epub 2022/12/17. doi: 10.3389/fonc.2022.1025270. PubMed PMID: 36523962; PubMed Central PMCID: PMCPMC9745808.

5. Muir T, Bertino G, Groselj A, Ratnam L, Kis E, Odili J, et al. Bleomycin electrosclerotherapy (BEST) for the treatment of vascular malformations. An International Network for Sharing Practices on Electrochemotherapy (InspECT) study group report. Radiol Oncol. 2023;57(2):141-9. Epub 2023/06/21. doi: 10.2478/raon-2023-0029. PubMed PMID: 37341196; PubMed Central PMCID: PMCPMC10286891.

6. Muir T, Wohlgemuth WA, Cemazar M, Bertino G, Groselj A, Ratnam LA, et al. Current Operating Procedure (COP) for Bleomycin ElectroScleroTherapy (BEST) of low-flow vascular malformations. Radiol Oncol. 2024;58(4):469-79. Epub 2024/11/28. doi: 10.2478/raon-2024-0061. PubMed PMID: 39608012; PubMed Central PMCID: PMCPMC11604259.

7. Schmidt VF, Masthoff M, Vielsmeier V, Seebauer CT, Cangir Ö, Meyer L, et al. Clinical Outcome and Quality of Life of Multimodal Treatment of Extracranial Arteriovenous Malformations: The APOLLON Study Protocol. Cardiovasc Intervent Radiol. 2022. Epub 2022/10/20. doi: 10.1007/s00270-022-03296-8. PubMed PMID: 36261507.

8. Filippiadis DK, Binkert C, Pellerin O, Hoffmann RT, Krajina A, Pereira PL. Cirse Quality Assurance Document and Standards for Classification of Complications: The Cirse Classification System. CardioVascular and Interventional Radiology. 2017;40(8):1141-6. doi: 10.1007/s00270-017-1703-4.

9. Wohlgemuth WA, Müller-Wille R, Meyer L, Wildgruber M, Guntau M, Heydt SV, et al. Bleomycin electrosclerotherapy in therapy-resistant venous malformations of the body. J Vasc Surg Venous Lymphat Disord. 2021;9(3):731-9. Epub 2020/10/13. doi: 10.1016/j.jvsv.2020.09.009. PubMed PMID: 33045393.

10. Cho SK, Do YS, Shin SW, Kim DI, Kim YW, Park KB, et al. Arteriovenous malformations of the body and extremities: analysis of therapeutic outcomes and approaches according to a modified angiographic classification. J Endovasc Ther. 2006;13(4):527-38. Epub 2006/08/25. doi: 10.1583/05-1769.1. PubMed PMID: 16928170.

11. Kohout MP, Hansen M, Pribaz JJ, Mulliken JB. Arteriovenous malformations of the head and neck: natural history and management. Plast Reconstr Surg. 1998;102(3):643-54. Epub 1998/09/04. doi: 10.1097/00006534-199809030-00006. PubMed PMID: 9727427.
